# Solving the general inter-ring distances optimization problem for concentric ring electrodes to improve Laplacian estimation

**DOI:** 10.1186/s12938-018-0549-6

**Published:** 2018-08-30

**Authors:** Oleksandr Makeyev

**Affiliations:** Department of Mathematics, Diné College, 1 Circle Dr, Tsaile, AZ 86556 USA

**Keywords:** Electrophysiology, Electroencephalography, Wearable sensors, Concentric ring electrodes, Laplacian, Optimization, Inter-ring distances, Finite element method, Modeling

## Abstract

**Background:**

Superiority of noninvasive tripolar concentric ring electrodes over conventional disc electrodes in accuracy of surface Laplacian estimation has been demonstrated in a range of electrophysiological measurement applications. Recently, a general approach to Laplacian estimation for an (*n* + 1)-polar electrode with *n* rings using the (4*n* + 1)-point method has been proposed and used to introduce novel multipolar and variable inter-ring distances electrode configurations. While only linearly increasing and linearly decreasing inter-ring distances have been considered previously, this paper defines and solves the general inter-ring distances optimization problem for the (4*n* + 1)-point method.

**Results:**

General inter-ring distances optimization problem is solved for tripolar (*n* = 2) and quadripolar (*n* = 3) concentric ring electrode configurations through minimizing the truncation error of Laplacian estimation. For tripolar configuration with middle ring radius *αr* and outer ring radius *r* the optimal range of values for *α* was determined to be 0 < *α* ≤ 0.22 while for quadripolar configuration with an additional middle ring with radius *βr* the optimal range of values for *α* and *β* was determined by inequalities 0 < *α* < *β* < 1 and *αβ* ≤ 0.21. Finite element method modeling and full factorial analysis of variance were used to confirm statistical significance of Laplacian estimation accuracy improvement due to optimization of inter-ring distances (*p* < 0.0001).

**Conclusions:**

Obtained results suggest the potential of using optimization of inter-ring distances to improve the accuracy of surface Laplacian estimation via concentric ring electrodes. Identical approach can be applied to solving corresponding inter-ring distances optimization problems for electrode configurations with higher numbers of concentric rings. Solutions of the proposed inter-ring distances optimization problem define the class of the optimized inter-ring distances electrode designs. These designs may result in improved noninvasive sensors for measurement systems that use concentric ring electrodes to acquire electrical signals such as from the brain, intestines, heart or uterus for diagnostic purposes.

## Background

**Fig. 1:**
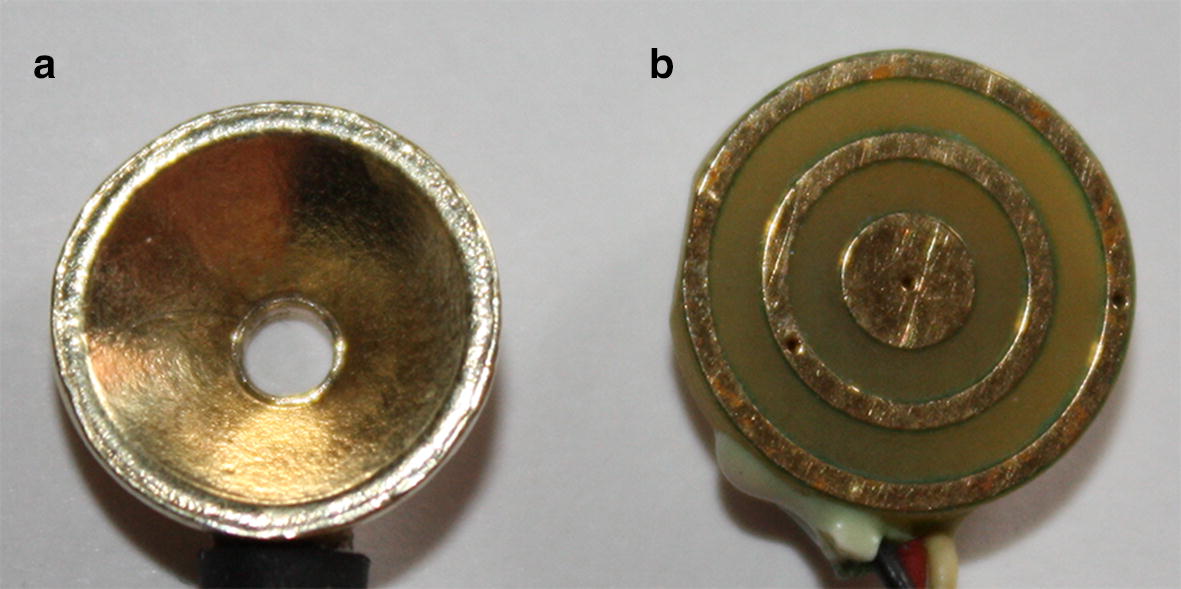
Conventional disc electrode (**a**) and tripolar concentric ring electrode (**b**)

Noninvasive concentric ring electrodes (CREs) have been shown to estimate the surface Laplacian, the second spatial derivative of the potentials on the scalp surface for the case of electroencephalogram (EEG), directly at each electrode instead of combining the data from an array of conventional, single pole, disc electrodes (Fig. 1a). In particular, tripolar CREs (TCREs; Fig. 1b) estimate the surface Laplacian using the nine-point method, an extension of the five-point method (FPM) used for bipolar CREs, and significantly better than other electrode systems including bipolar and quasi-bipolar CRE configurations [1, 2]. Compared to EEG via disc electrodes Laplacian EEG via TCREs (tEEG) has been demonstrated to have significantly better spatial selectivity (approximately 2.5 times higher), signal-to-noise ratio (approximately 3.7 times higher), and mutual information (approximately 12 times lower) [3]. Thanks to these properties TCREs found numerous applications in a wide range of areas where electrical signals from the brain are measured including brain–computer interface [4, 5], seizure onset detection [6, 7], detection of high-frequency oscillations and seizure onset zones [8], etc. Review of recent advances in high-frequency oscillations and seizure onset detection based on tEEG via TCREs is available in [9]. These EEG related applications of TCREs along with recent CRE applications related to electroenterograms [10, 11], electrocardiograms (ECG) [12–15], and electrohysterograms [16] suggest the potential of CRE technology in noninvasive electrophysiological measurement.

In order to further improve the CRE design several approaches were proposed including printing disposable CREs on flexible substrates to increase the electrode’s ability to adjust to body contours for better contact and to provide higher signal amplitude and signal-to-noise ratio [11, 13, 15, 16]. Other approaches concentrate on assessing the effect of ring dimensions [14, 15] and electrode position [14] on recorded signal and making the measurement system wireless [15]. However, the signal recorded from CREs in [11, 13–16] is either a surface Laplacian estimated for the case of the outer ring and the central disc of the TCRE being shorted together (quasi-bipolar CRE configuration) or a set of bipolar signals representing differences between potentials recorded from the rings and the central disc. Alternatively, signals from all the recording surfaces of each TCRE can be combined into a surface Laplacian estimate signal similar to tEEG. Previously, this approach has resulted in significantly higher Laplacian estimation accuracy and radial attenuation for TCREs compared to bipolar and quasi-bipolar CRE configurations [1, 2]. This inspired the recent efforts to further improve the Laplacian estimation accuracy via CREs by increasing the number of concentric rings [17] and varying the inter-ring distances (distances between consecutive rings) [18] described below.

In [17] a general approach to estimation of the Laplacian for an (*n* + 1)-polar electrode with *n* rings using the (4*n* + 1)-point method for *n* ≥ 2 has been proposed. This method allows cancellation of all the Taylor series truncation terms up to the order of 2*n* which has been shown to be the highest order achievable for a CRE with *n* rings [17]. In [17] (4*n* + 1)-point method was used to demonstrate that accuracy of Laplacian estimation can be improved with an increase of the number of rings, *n,* by proposing multipolar CRE configurations. Such configurations with *n* equal to up to 6 rings (septapolar electrode configuration) were compared using finite element method (FEM) modeling and the obtained results suggested statistical significance (*p* < 0.0001) of the increase in Laplacian accuracy due to an increase of *n* [17]. In [18] (4*n* + 1)-point method was used to demonstrate that accuracy of the Laplacian estimation can be improved with transitioning from the previously used constant inter-ring distances by proposing novel variable inter-ring distances CRE configurations. Laplacian estimates for linearly increasing and linearly decreasing inter-ring distances TCRE (*n* = 2) and quadripolar CRE (QCRE; *n* = 3) configurations were directly compared to their constant inter-ring distances counterparts using analytic analysis and FEM modeling. The main results included establishing a connection between the analytic truncation term coefficient ratios from the Taylor series used in (4*n* + 1)-point method and respective ratios of Laplacian estimation errors computed using the FEM model [18]. Both analytic and FEM results were consistent in suggesting that CRE configurations with linearly increasing inter-ring distances may offer more accurate Laplacian estimates compared to CRE configurations with constant inter-ring distances. In particular, for TCREs the Laplacian estimation error may be decreased more than twofold while for QCREs more than a sixfold decrease in estimation error is expected [18]. First physical TCRE prototypes closely resembling the proposed increasing inter-ring distances TCRE design (physical TCRE prototype has a 4:7 ratio of inter-ring distances compared to the 1:2 ratio in the increasing inter-ring distances design proposed in [18]) were assessed in [19] on human EEG, ECG, and electromyogram (EMG) data with promising results.

One of the limitations of [18] was that only linearly variable inter-ring distances were considered while it was hypothesized that optimal inter-ring distances are likely to have a nonlinear relationship. In this paper, the general inter-ring distances optimization problem for the (4*n* + 1)-point method of Laplacian estimation is proposed and solved for TCRE and QCRE configurations. The main results include determining the ranges of optimal distances between the central disc and the concentric rings that allow minimizing the truncation error of Laplacian estimation through minimizing the absolute values of truncation term coefficients to be within the 5th percentile. For TCRE with middle ring radius *αr* and outer ring radius *r* the optimal range of values for coefficient *α* was determined to be 0 < *α* ≤ 0.22 while for QCRE with the first middle ring radius *αr*, the second middle ring radius *βr*, and the outer ring radius *r* the optimal range of values for coefficients *α* and *β* was determined to be defined by inequalities 0 < *α *<* β *<1 and *αβ *≤ 0.21. Truncation term coefficient functions used to solve the general inter-ring distances optimization problem have been validated using ratios of truncation term coefficients for constant and linearly variable inter-ring distances TCRE and QCRE configurations from [18].

Moreover, while in [17] the analysis of variance (ANOVA) has been performed for multipolar CREs to confirm the statistical significance of obtained FEM results, no such analysis has been performed in [18] for variable inter-ring distances CREs. Even after it was added in [20] it lacked factor levels corresponding to optimized inter-ring distances CREs. In this paper, a full factorial design of ANOVA is performed on FEM data that included optimized inter-ring distances CRE configurations to assess statistical significance of the effect of optimization of inter-ring distances on accuracy of Laplacian estimation.

This paper is organized as follows: notations and preliminaries including basic case of FPM as well as the general (4*n* + 1)-point method of surface Laplacian estimation for (*n* + 1)-polar CRE with *n* rings are presented in “Methods” section. This section also contains derivation of the truncation term coefficient functions for TCRE and QCRE configurations and defines the general inter-ring distances optimization problem as a constrained optimization problem to minimize the absolute values of truncation term coefficients using the derived truncation term coefficient functions. Finally, FEM model and full factorial ANOVA design are presented. Main results including validation of the proposed truncation term coefficient functions using the ratios of truncation term coefficients for constant and linearly variable inter-ring distances TCRE and QCRE configurations from [18] and solving the proposed general inter-ring distances optimization problem for TCRE and QCRE configurations are presented in “Results” section along with FEM modeling and ANOVA results. Discussion of the obtained results and directions of future work are presented in “Discussion” section followed by the overall conclusions.

## Methods

### Notations and preliminaries

**Fig. 2:**
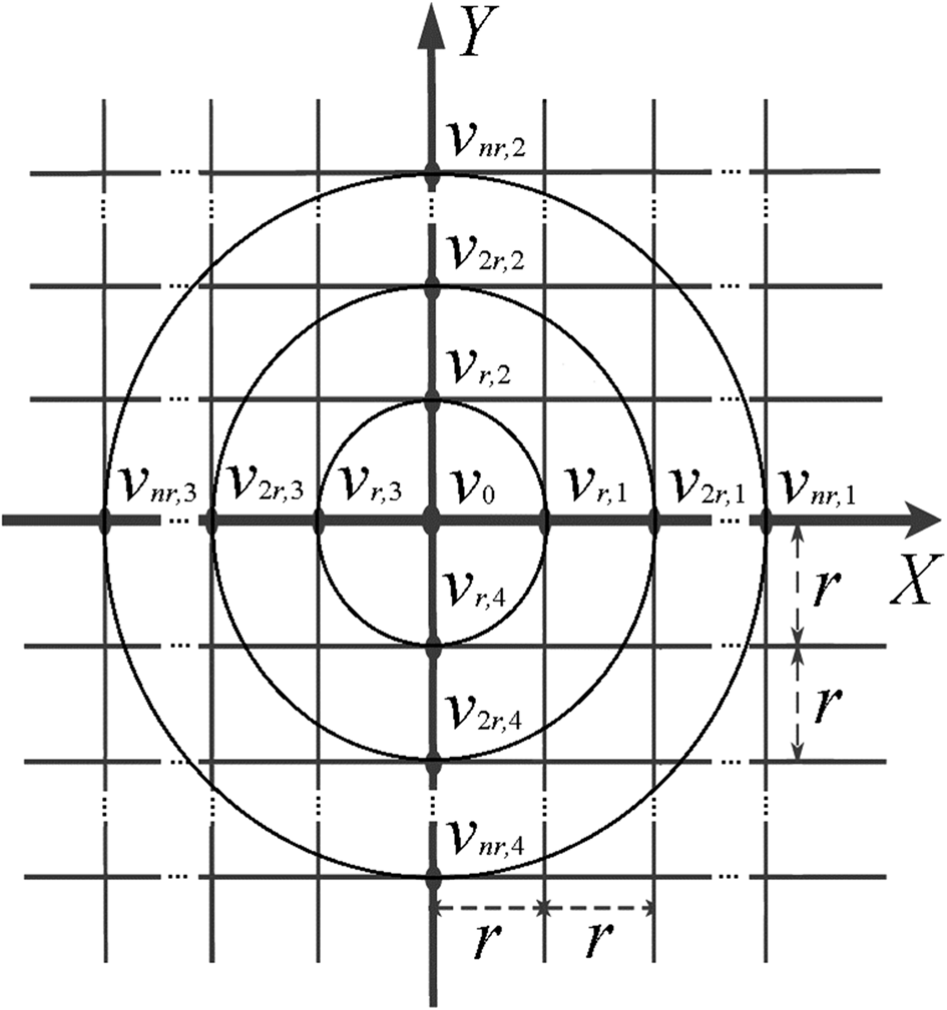
Regular plane square grid with inter-point distances equal to *r*

In [17] the general (4*n* + 1)-point method for constant inter-ring distances (*n* + 1)-polar CRE with *n* rings was proposed. It was derived using a regular plane square grid with all inter-point distances equal to *r* presented in Fig. 2.

First, FPM was applied to the points with potentials *v*_0_, *v*_*r*,1_, *v*_*r*,2_, *v*_r,3_, and *v*_*r*,4_ (Fig. 2) following Huiskamp’s calculation of the Laplacian potential ∆*v*_0_ using Taylor series [21]:

1 $$\Delta v_{0} = \frac{{d^{2} v}}{{dx^{2} }} + \frac{{d^{2} v}}{{dy^{2} }} = \frac{1}{{r^{2} }}\left( {\sum\limits_{i = 1}^{4} {v_{r,i} - 4} v_{0} } \right) + O\left( {r^{2} } \right)$$

where $O\left( {r^{2} } \right) = \frac{{r^{2} }}{4!}\left( {\frac{{d^{4} v}}{{dx^{4} }} + \frac{{d^{4} v}}{{dy^{4} }}} \right) + \frac{{r^{4} }}{6!}\left( {\frac{{d^{6} v}}{{dx^{6} }} + \frac{{d^{6} v}}{{dy^{6} }}} \right) + \cdots$ is the truncation error.

Equation (1) can be generalized by taking the integral along the circle of radius *r* around the point with potential *v*_0_. Defining *x* = *r*cos(*θ*) and *y* = *r*sin(*θ*) as in Huiskamp [21] we obtain:

2 $$\frac{1}{2\pi }\int\limits_{0}^{2\pi } {v\left( {r,\theta } \right)d\theta - v_{0} = } \frac{{r^{2} }}{4}\Delta v_{0} + \frac{{r^{4} }}{4!}\int\limits_{0}^{2\pi } {\sum\limits_{j = 0}^{4} {\sin^{4 - j} \left( \theta \right)\cos^{j} \left( \theta \right)d\theta \left( {\frac{{d^{4} v}}{{dx^{4 - j} dy^{j} }}} \right)} } + \cdots$$

where $\frac{1}{2\pi }\int\nolimits_{0}^{2\pi } {v\left( {r,\theta } \right)d\theta }$ is the average potential on the ring of radius *r* and *v*_0_ is the potential on the central disc of the CRE.

Next, for the case of multipolar CRE with *n* rings (*n* ≥ 2), we consider a set of *n* FPM equations. Each equation corresponds to one of the *n* rings with ring radii ranging from *r* to *nr*. These equations are derived in a manner identical to the way the FPM equation for the ring of radius *r* has been derived in Eq. (2). For example, we obtain the FPM equation for the ring of radius *nr* (points with potentials *v*_0_, *v*_*nr*,1_, *v*_*nr*,2_, *v*_*nr*,3_, and *v*_*nr*,4_ in Fig. 2) as follows:

3 $$\begin{aligned} \frac{1}{2\pi }\int\limits_{0}^{2\pi } {v\left( {nr,\theta } \right)d\theta - v_{0} =\, } \frac{{\left( {nr} \right)^{2} }}{4}\Delta v_{0} + \frac{{\left( {nr} \right)^{4} }}{4!}\int\limits_{0}^{2\pi } {\sum\limits_{j = 0}^{4} {\sin^{4 - j} \left( \theta \right)\cos^{j} \left( \theta \right)\,\,d\theta \left( {\frac{{d^{4} v}}{{dx^{4 - j} dy^{j} }}} \right)} } \hfill \\ + \frac{{\left( {nr} \right)^{6} }}{6!}\int\limits_{0}^{2\pi } {\sum\limits_{j = 0}^{6} {\sin^{6 - j} \left( \theta \right)\,\,\cos^{j} \left( \theta \right)d\theta \left( {\frac{{d^{6} v}}{{dx^{6 - j} dy^{j} }}} \right)} } + \cdots \hfill \\ \end{aligned}$$

where $\frac{1}{2\pi }\int\nolimits_{0}^{2\pi } {v\left( {nr,\theta } \right)d\theta }$ is the average potential on the ring of radius *nr* and *v*_0_ is the potential on the central disc of the CRE.

Finally, to estimate the Laplacian, the *n* equations, representing differences between average potentials on the *n* rings and the potential on the central disc of the CRE, are linearly combined in a way that cancels all the Taylor series truncation terms up to the order of 2*n*. To obtain such linear combination, the coefficients *l*^*k*^ of the truncation terms with the general form $\frac{{\left( {lr} \right)^{k} }}{k!}\int\nolimits_{0}^{2\pi } {\sum\nolimits_{j = 0}^{k} {\sin^{k - j} \left( \theta \right)\cos^{j} \left( \theta \right)d\theta \left( {\frac{{d^{k} v}}{{dx^{k - j} dy^{j} }}} \right)} }$ for even order *k* ranging from 4 to 2*n* and ring radius multiplier *l* ranging from 1 [Eq. (2)] to *n* [Eq. (3)] are arranged into an *n − *1 by *n* matrix *A* that is a function only of the number of the rings *n*:

4 $$A = \left( {\begin{array}{*{20}c} {1^{4} } & {2^{4} } & \cdots & {n^{4} } \\ {1^{6} } & {2^{6} } & \cdots & {n^{6} } \\ \vdots & \vdots & \ddots & \vdots \\ {1^{2n} } & {2^{2n} } & \cdots & {n^{2n} } \\ \end{array} } \right) = \left( {\begin{array}{*{20}c} 1 & {2^{4} } & \cdots & {n^{4} } \\ 1 & {2^{6} } & \cdots & {n^{6} } \\ \vdots & \vdots & \ddots & \vdots \\ 1 & {2^{2n} } & \cdots & {n^{2n} } \\ \end{array} } \right)$$

The null space (or kernel) of matrix *A* is an *n*-dimensional vector $\bar{x} = \left( {x_{1} ,\;x_{2} ,\; \ldots ,\;x_{n} } \right)$ that is a nontrivial solution of a matrix equation $A\bar{x} = \bar{0}$. The dot product of $\bar{x}$ and a vector consisting of *n* coefficients *l*^*k*^ corresponding to all the ring radii [i.e. $\left( {1,\;2^{k} ,\; \ldots ,\;n^{k} } \right)$] for all even orders *k* ranging from 4 to 2*n* is equal to 0:

5 $$x_{1} + 2^{k} x_{2} + \; \cdots + n^{k} x_{n} = 0$$

This allows cancellation of all the truncation terms up to the order of 2*n* when the Laplacian estimate is calculated as the linear combination of equations representing differences of potentials from each of the *n* rings and the central disc ranging from Eq. (2) for the first, innermost concentric ring and up to Eq. (3) for the *n*-th, outermost concentric ring. The null space vector $\bar{x}$ is used as coefficients and the linear combination is solved for the Laplacian ∆*v*_0_:

6 $$\Delta v_{0} \cong \frac{4}{{r^{2} \left( {x_{1} + \cdots + n^{2} x_{n} } \right)}}\left[ {x_{1} \left( {\frac{1}{2\pi }\int\limits_{0}^{2\pi } {v(r,\theta )d\theta - v_{0} } } \right)} \right.\left. { + \cdots + x_{n} \left( {\frac{1}{2\pi }\int\limits_{0}^{2\pi } {v(nr,\theta )d\theta - v_{0} } } \right)} \right]$$

This Laplacian estimate signal is calculated using a custom preamplifier board and is the only signal sent to the clinical amplifier for each CRE.

Finally, in [18] (4*n* + 1)-point method from [17] has been modified to accommodate CRE configurations with variable inter-ring distances that increase or decrease linearly the further the concentric ring lies from the central disc. In both cases sums of all the inter-ring distances to the outermost, *n*-th, ring were calculated using the formula for the *n*-th term of the triangular number sequence equal to *n*(*n* + 1)/2 [22]. Consequently, matrix *A* of truncation term coefficients *l*^*k*^ from Eq. (4) has been modified for linearly increasing (*A*′) and linearly decreasing (*A*′′) inter-ring distances CREs respectively [18]:

7 $$A^{\prime} = \left( {\begin{array}{*{20}c} 1 & {3^{4} } & \cdots & {\left( {\frac{{n\left( {n + 1} \right)}}{2}} \right)^{4} } \\ 1 & {3^{6} } & \cdots & {\left( {\frac{{n\left( {n + 1} \right)}}{2}} \right)^{6} } \\ \vdots & \vdots & \ddots & \vdots \\ 1 & {3^{2n} } & \cdots & {\left( {\frac{{n\left( {n + 1} \right)}}{2}} \right)^{2n} } \\ \end{array} } \right)$$

8 $$A^{\prime\prime} = \left( {\begin{array}{*{20}c} {n^{4} } & {\left( {2n - 1} \right)^{4} } & \cdots & {\left( {\frac{{n\left( {n + 1} \right)}}{2}} \right)^{4} } \\ {n^{6} } & {\left( {2n - 1} \right)^{6} } & \cdots & {\left( {\frac{{n\left( {n + 1} \right)}}{2}} \right)^{6} } \\ \vdots & \vdots & \ddots & \vdots \\ {n^{2n} } & {\left( {2n - 1} \right)^{2n} } & \cdots & {\left( {\frac{{n\left( {n + 1} \right)}}{2}} \right)^{2n} } \\ \end{array} } \right)$$

Instead of continuing to modify matrix *A* to assess any additional modalities of variable inter-ring distances CREs (including nonlinear ones) the way it was done in [18] resulting in Eqs. (7) and (8), in this paper the general inter-ring distances optimization problem for the (4*n* + 1)-point method of Laplacian estimation is solved for TCRE and QCRE configurations.

### Truncation term coefficient function for the TCRE configuration

**Fig. 3:**
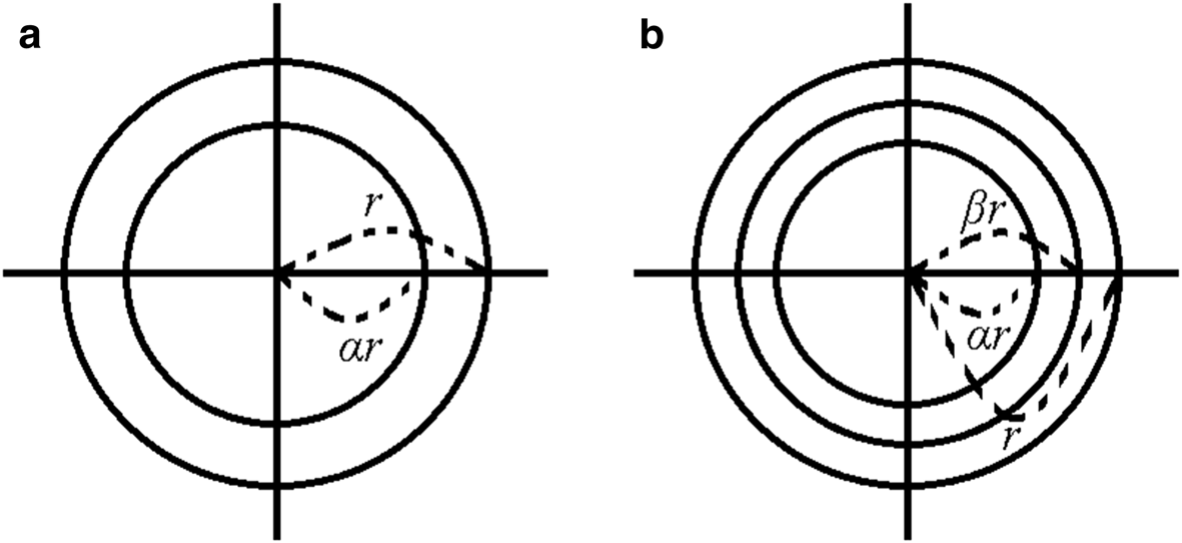
TCRE (**a**) and QCRE (**b**) configuration setup

Assuming that our TCRE (*n* = 2) has two rings with radii *αr* and *r* where coefficient *α* satisfies 0 < *α *<1 (Fig. 3a), for each ring the integral of the Taylor series is taken along the circle with the corresponding radius. For the ring with radius *r* we obtain Eq. (2) while for the ring with radius *αr* we obtain:

9 $$\begin{aligned} \frac{1}{2\pi }\int\limits_{0}^{2\pi } {v\left( {\alpha r,\theta } \right)d\theta = v_{0} + } \frac{{\left( {\alpha r} \right)^{2} }}{4}\Delta v_{0} + \frac{{\left( {\alpha r} \right)^{4} }}{4!}\int\limits_{0}^{2\pi } {\sum\limits_{j = 0}^{4} {\sin^{4 - j} \left( \theta \right)\cos^{j} \left( \theta \right)d\theta } } \left( {\frac{{d^{4} v}}{{dx^{4 - j} dy^{j} }}} \right) \hfill \\ + \frac{{\left( {\alpha r} \right)^{6} }}{6!}\int\limits_{0}^{2\pi } {\sum\limits_{j = 0}^{6} {\sin^{6 - j} \left( \theta \right)\cos^{j} \left( \theta \right)d\theta } } \left( {\frac{{d^{6} v}}{{dx^{6 - j} dy^{j} }}} \right) + \cdots \hfill \\ \end{aligned}$$

For this generalized TCRE setup, modified matrix *A* of truncation term coefficients *l*^*k*^ from Eq. (4) becomes:

10 $$A^{TCRE} = \left( {\alpha^{4} \quad 1^{4} } \right) = \left( {\alpha^{4} \quad 1} \right)$$

The null space of *A*^*TCRE*^, $\bar{x}^{TCRE}$, is equal up to (multiplication by) a constant factor to:

11 $$\bar{x}^{TCRE} = \left( { - \frac{1}{{\alpha^{4} }},\;1} \right)$$

Null space vectors such as $\bar{x}^{TCRE}$ from (11) are not unique. From the properties of matrix multiplication it follows that for any vector $\bar{x}^{TCRE}$ that belongs to the null space of matrix *A*^*TCRE*^ and a constant factor *c* the scaled vector $c\bar{x}^{TCRE}$ also belongs to the null space of matrix *A*^*TCRE*^ since $A^{TCRE} (c\bar{x}^{TCRE} ) = c(A^{TCRE} \bar{x}^{TCRE} ) = c\bar{0} = \bar{0}$.

We combine Eqs. (9) and (2) using the null space vector $\bar{x}^{TCRE}$ from Eq. (11) as coefficients by multiplying Eq. (9) by − 1/*α*^4^, multiplying Eq. (2) by 1, and adding the two resulting products together with the sum being solved for the Laplacian ∆*v*_0_:

12 $$\Delta v_{0} = \frac{4}{{r^{2} \left( {1 - \frac{1}{{\alpha ^{2} }}} \right)}}\left[ { - \frac{1}{{\alpha ^{4} }}\left( {v_{{MR}} - v_{0} } \right) + \left( {v_{{OR}} - v_{0} } \right) + \sum\limits_{{k = 6,8, \ldots }}^{\infty } {\frac{{\left( {1 - \alpha ^{{k - 4}} } \right)r^{k} }}{{k!}}\int\limits_{0}^{{2\pi }} {\sum\limits_{{j = 0}}^{k} {\sin ^{{k - j}} \left( \theta \right)\cos ^{j} \left( \theta \right)d\theta } } \left( {\frac{{\partial ^{k} v}}{{\partial x^{{k - j}} \partial y^{j} }}} \right)} } \right]$$

where $v_{MR} = \frac{1}{2\pi }\int\nolimits_{0}^{2\pi } {v\left( {\alpha r,\theta } \right)d\theta }$ is the potential on the middle ring of the radius *αr* and $v_{OR} = \frac{1}{2\pi }\int\nolimits_{0}^{2\pi } {v\left( {r,\theta } \right)d\theta }$ is the potential on the outer ring of the radius *r*.

The Laplacian estimate from Eq. (12) allows cancellation of the fourth (2*n* = 4 for *n* = 2) order truncation term. After simplification, the coefficients *c*^*TCRE*^(*α*, *k*) of truncation terms with the general form $\frac{{c^{TCRE} \left( {\alpha ,k} \right)r^{k - 2} }}{k!}\int\nolimits_{0}^{2\pi } {\sum\nolimits_{j = 0}^{k} {\sin^{k - j} \left( \theta \right)\,\,\cos^{j} \left( \theta \right)d\theta } } \left( {\frac{{\partial^{k} v}}{{\partial x^{k - j} \partial y^{j} }}} \right)$ can be expressed as the function of coefficient *α* and the truncation term order *k* for even *k* ≥ 6:

13 $$c^{TCRE} \left( {\alpha ,k} \right) = \frac{{4\;\left( {\alpha^{4} - \alpha^{k} } \right)}}{{\alpha^{2} \left( {\alpha^{2} - 1} \right)}}$$

### Truncation term coefficient function for the QCRE configuration

Assuming that our QCRE (*n* = 3) has three rings with radii *αr*, *βr*, and *r* where coefficients *α* and *β* satisfy 0 < *α *<* β *<1 (Fig. 3b), for each ring the integral of the Taylor series is taken along the circle with the corresponding radius. For the ring with radius *r* we obtain Eq. (2), for the ring with radius *αr* we obtain Eq. (9), and for the ring with radius *βr* we obtain:

14 $$\begin{aligned} \frac{1}{2\pi }\int\limits_{0}^{2\pi } {v\left( {\beta r,\theta } \right)d\theta = v_{0} + } \frac{{\left( {\beta r} \right)^{2} }}{4}\Delta v_{0} + \frac{{\left( {\beta r} \right)^{4} }}{4!}\int\limits_{0}^{2\pi } {\sum\limits_{j = 0}^{4} {\sin^{4 - j} \left( \theta \right)\cos^{j} \left( \theta \right)d\theta } } \left( {\frac{{d^{4} v}}{{dx^{4 - j} dy^{j} }}} \right) \hfill \\ + \frac{{\left( {\beta r} \right)^{6} }}{6!}\int\limits_{0}^{2\pi } {\sum\limits_{j = 0}^{6} {\sin^{6 - j} \left( \theta \right)\cos^{j} \left( \theta \right)d\theta } } \left( {\frac{{d^{6} v}}{{dx^{6 - j} dy^{j} }}} \right) + \cdots \hfill \\ \end{aligned}$$

For this generalized QCRE setup, modified matrix *A* of truncation term coefficients *l*^*k*^ from Eq. (4) becomes:

15 $$A^{QCRE} = \left( \begin{aligned} \begin{array}{*{20}c} {\alpha^{4} } & {\beta^{4} } & {1^{4} } \\ \end{array} \hfill \\ \begin{array}{*{20}c} {\alpha^{6} } & {\beta^{6} } & {1^{6} } \\ \end{array} \hfill \\ \end{aligned} \right) = \left( \begin{aligned} \begin{array}{*{20}c} {\alpha^{4} } & {\beta^{4} } & 1 \\ \end{array} \hfill \\ \begin{array}{*{20}c} {\alpha^{6} } & {\beta^{6} } & 1 \\ \end{array} \hfill \\ \end{aligned} \right)$$

The null space of *A*^*QCRE*^, $\bar{x}^{QCRE}$, is equal up to a (multiplication by) a constant factor to:

16 $$\bar{x}^{QCRE} = \left( { - \frac{{1 - \beta^{2} }}{{\alpha^{4} \left( {\alpha^{2} - \beta^{2} } \right)}},\; - \frac{{\alpha^{2} - 1}}{{\beta^{4} \left( {\alpha^{2} - \beta^{2} } \right)}},1} \right)$$

We combine Eqs. (2), (9), and (14) using the null space vector $\bar{x}^{QCRE}$ from Eq. (16) as coefficients by multiplying Eq. (9) by $- \frac{{1 - \beta^{2} }}{{\alpha^{4} \left( {\alpha^{2} - \beta^{2} } \right)}}$, multiplying Eq. (14) by $- \frac{{\alpha^{2} - 1}}{{\beta^{4} \left( {\alpha^{2} - \beta^{2} } \right)}}$, multiplying Eq. (2) by 1, and adding the three resulting products together with the sum being solved for the Laplacian ∆*v*_0_. Such a Laplacian estimate allows cancellation of the fourth and the sixth (2*n* = 6 for *n* = 3) order truncation terms. It can be shown that, after simplification, the coefficients *c*^*QCRE*^(*α*, *β*, *k*) of truncation terms with the general form $\frac{{c^{QCRE} \left( {\alpha ,\beta ,k} \right)r^{k - 2} }}{k!}\int\nolimits_{0}^{2\pi } {\sum\nolimits_{j = 0}^{k} {\sin^{k - j} \left( \theta \right)\cos^{j} \left( \theta \right)d\theta } } \left( {\frac{{\partial^{k} v}}{{\partial x^{k - j} \partial y^{j} }}} \right)$ can be expressed as the function of coefficients *α* and *β* and the truncation term order *k* for even *k* ≥ 8:

17 $$c^{QCRE} \left( {\alpha ,\beta ,k} \right) = \frac{{4\left[ {\alpha^{k} \beta^{4} \left( {\beta^{2} - 1} \right) + \alpha^{6} \left( {\beta^{4} - \beta^{k} } \right) + \alpha^{4} \left( {\beta^{k} - \beta^{6} } \right)} \right]}}{{\alpha^{2} \beta^{2} \left( {\alpha^{2} - 1} \right)\left( {\beta^{2} - 1} \right)\left( {\alpha^{2} - \beta^{2} } \right)}}$$

### General inter-ring distances optimization problem and its constraints

A constrained optimization problem is proposed to minimize the absolute values of truncation term coefficients for TCRE and QCRE configurations using functions *c*^*TCRE*^(*α*, *k*) and *c*^*QCRE*^(*α*, *β*, *k*) from Eqs. (13) and (17) respectively. Solving this problem will result in optimized inter-ring distances TCRE and QCRE designs that minimize the truncation error and, therefore, maximize the accuracy of surface Laplacian estimates. Absolute values of truncation term coefficients are used since the signs of the truncation term coefficients have been shown in [18] to be consistent for both constant and variable inter-ring distances CRE configurations: all negative for TCREs and all positive for QCREs. Therefore, for both configurations larger absolute values of truncation term coefficients will translate into larger truncation error. The optimization problem is solved for the lowest nonzero truncation term order equal to 6 and 8 for TCRE and QCRE configurations respectively as the ones that contribute the most to the truncation error since according to [23] for Taylor series “higher-order terms usually contribute negligibly to the final sum and can be justifiably discarded.” Formal definitions of the optimization problem for TCRE and QCRE configurations are $\mathop{\text{min} } \limits _{0 < \alpha < 1} \left| {c^{TCRE} \left( {\alpha ,6} \right)} \right|$ and $\mathop {\hbox{min} }\limits_{0 < \alpha < \beta < 1} \left| {c^{QCRE} \left( {\alpha ,\beta ,8} \right)} \right|$ respectively.

The algorithm of finding global solution to this constrained optimization problem is based on using the 5th percentile to determine the boundary values separating the lowest 5% from the highest 95% of the absolute values of truncation term coefficients. Absolute values of truncation term coefficients within the 5th percentile determine the range of optimal distances between the central disc and the concentric rings to be used in the optimized inter-ring distances TCRE and QCRE designs.

### FEM modeling

To directly compare the surface Laplacian estimates for constant inter-ring distances TCRE and QCRE configurations to their counterparts with variable (including optimized) inter-ring distances a FEM model from [17, 18] was used. Evenly spaced square mesh size of 5000 × 5000 was located in the first quadrant of the *X*–*Y* plane above a unit charge dipole projected to the center of the mesh and oriented towards the positive direction of the *Z* axis. Comparisons to the linearly increasing [18] and novel quadratically increasing inter-ring distances TCRE and QCRE configurations respectively were drawn. In the novel quadratically increasing CRE configurations the inter-ring distances are increasing as a quadratic function *f*(*s*) = *s*^2^ rather than as a linear identity function *f*(*s*) = *s* of the concentric ring number *s* counting from the central disc. Bipolar CRE configuration (*n* = 1) was also included into the FEM model. Matlab (Mathworks, Natick, MA, USA) was used for all the FEM modeling.

At each point of the mesh, the electric potential was generated by a unity dipole at depth equal to 3 cm. The medium was assumed to be homogeneous with the conductivity of 7.14 mS/cm to emulate biological tissue [24]. The analytical Laplacian was then calculated at each point of the mesh, by taking the second derivative of the electric potential [17, 18]. Laplacian estimates for different CRE configurations were computed at each point of the mesh where appropriate boundary conditions could be applied for different CRE diameters. Laplacian estimate coefficients for constant inter-ring distances CRE configurations were previously derived using the null space of matrix *A* from Eq. (4): (16, − 1) for TCRE and (270, − 27, 2) for QCRE [17]. Coefficients for linearly increasing inter-ring distances CRE configurations were previously derived using the null space of matrix *A’* from Eq. (7): (81, − 1) for TCRE and (4374, –70, 1) for QCRE [18]. Derivation of Laplacian estimate coefficients for novel quadratically increasing inter-ring distances CRE configurations was performed using generalized null space equations proposed in this paper. For the TCRE configuration Eq. (11) was used for *α* = 1/5 to obtain coefficients (625, − 1) while for the QCRE configuration (16) was used for *α* = 1/14 and *β* = 5/14 to obtain coefficients (34,214,250, − 62,426, 125). These seven Laplacian estimates including three for TCREs (with constant, linearly increasing, and quadratically increasing inter-ring distances respectively), three for QCREs, and one for the bipolar CRE configuration were then compared with the calculated analytical Laplacian for each point of the mesh where corresponding Laplacian estimates were computed using Relative Error and Maximum Error measures [17, 18]:

18 $${\text{Relative Error}}^{i} = \sqrt {\frac{{\sum {(\Delta v - } \Delta^{i} v)^{2} }}{{\sum {(\Delta v)^{2} } }}}$$

19 $${\text{Maximum Error}}^{i} = \hbox{max} \left| {\Delta v - \Delta^{i} v} \right|$$

where *i* represents seven CRE configurations, ∆^*i*^*v* represents their corresponding Laplacian estimates, and ∆*v* represents the analytical Laplacian potential. More detail on the FEM model used can be found in [17, 18].

Design-Expert (Stat-Ease Inc., Minneapolis, MN, USA) was used for all the statistical analysis of FEM modeling results. Full factorial ANOVA was used with one categorical and two numerical factors [25]. The categorical factor (A) was the inter-ring distances of the CRE presented at three levels corresponding to electrodes with constant inter-ring distances, linearly increasing inter-ring distances, and novel quadratically increasing inter-ring distances respectively. The first numerical factor (B) was the number of concentric rings in the CRE presented at two levels corresponding to TCRE (two concentric rings) and QCRE (three concentric rings) configurations. The second numerical factor (C) was the CRE diameter presented at ten levels uniformly distributed in the range from 0.5 to 5 cm. One possible nuisance factor is the type of the FEM model used in this study which is known but uncontrollable [25]. Two response variables were the Relative Error and Maximum Error of Laplacian estimation computed using Eqs. (18) and (19) respectively for each of the 3 × 2 × 10 = 60 combinations of levels for the three factors. Assumptions of ANOVA including normality, homogeneity of variance, and independence of observations were verified ensuring the validity of the analysis with no studentized residuals being outliers (falling outside of the [− 3, 3] range) [25]. Due to the deterministic nature of the FEM model randomizing the order of runs and adding replications were not feasible.

## Results

### Validating truncation term coefficient functions using ratios of truncation term coefficients for constant and linearly variable inter-ring distances TCRE and QCRE configurations

In [18] two special cases of variable inter-ring distances CREs: linearly increasing [Eq. (7)] and linearly decreasing [Eq. (8)] configurations were proposed and assessed. These two special cases were compared to constant inter-ring distances CREs. It was hypothesized that the ratios of constant inter-ring distances truncation term coefficients over the increasing inter-ring distances truncation term coefficients as well as the ratios of decreasing inter-ring distances truncation term coefficients over constant inter-ring distances truncation term coefficients calculated for TCRE and QCRE configurations will be comparable to the respective ratios of Relative and Maximum Errors of Laplacian estimation obtained using the FEM model. For constant inter-ring distances over increasing inter-ring distances, the truncation term coefficient ratios for the lowest nonzero truncation term for TCRE (sixth order) and QCRE (eighth order) configurations were calculated to be equal to 2.25 and 7.11 respectively which were comparable (difference of less than 5%) to the corresponding ratios of Relative and Maximum Errors obtained using the FEM model for TCRE (2.23 ± 0.02 and 2.22 ± 0.03 respectively) and QCRE (6.95 ± 0.14 and 6.91 ± 0.16) configurations [18]. For decreasing inter-ring distances over constant inter-ring distances, the coefficient truncation term coefficient ratios for the lowest nonzero truncation term for TCRE and QCRE configurations were calculated to be equal to 1.78 and 3.52 respectively which also were comparable (difference of less than 5%) to the corresponding ratios of Relative and Maximum Errors obtained using the FEM model for TCRE (1.75 ± 0.02 and 1.74 ± 0.03 respectively) and QCRE (3.41 ± 0.09 and 3.38 ± 0.11) configurations [18].

Without the truncation term coefficient functions from the general inter-ring distances optimization problem proposed in this study, in [18] all of the aforementioned analytic ratios had to be calculated independently from separate CRE setups while now they can be calculated using functions *c*^*TCRE*^(*α*, *k*) and *c*^*QCRE*^(*α*, *β*, *k*) from Eqs. (13) and (17) respectively. For constant inter-ring distances TCRE and QCRE configurations we have functions $c^{TCRE} \left( {\frac{1}{2},k} \right)$ and $c^{QCRE} \left( {\frac{1}{3},\frac{2}{3},k} \right)$ respectively. For linearly increasing inter-ring distances TCRE and QCRE configurations we have functions $c^{TCRE} \left( {\frac{1}{3},k} \right)$ and $c^{QCRE} \left( {\frac{1}{6},\frac{1}{2},k} \right)$ respectively. For linearly decreasing inter-ring distances TCRE and QCRE configurations we have functions $c^{TCRE} \left( {\frac{2}{3},k} \right)$ and $c^{QCRE} \left( {\frac{1}{2},\frac{5}{6},k} \right)$ respectively.

To validate the proposed functions *c*^*TCRE*^(*α*, *k*) and *c*^*QCRE*^(*α*, *β*, *k*) from Eqs. (13) and (17) respectively, the aforementioned analytic ratios (2.25, 7.11, 1.78, and 3.52) of truncation term coefficients from [18] were recalculated for the lowest nonzero truncation term orders equal to 6 and 8 for TCREs and QCREs respectively and rounded to the nearest hundredth:

20 $$\frac{{c^{TCRE} \left( {\frac{1}{2},6} \right)}}{{c^{TCRE} \left( {\frac{1}{3},6} \right)}} = \frac{ - 1}{{ - \frac{4}{9}}} = 2.25$$

21 $$\frac{{c^{QCRE} \left( {\frac{1}{3},\frac{2}{3},8} \right)}}{{c^{QCRE} \left( {\frac{1}{6},\frac{1}{2},8} \right)}} = \frac{{\frac{16}{81}}}{{\frac{1}{36}}} = 7.11$$

22 $$\frac{{c^{TCRE} \left( {\frac{2}{3},6} \right)}}{{c^{TCRE} \left( {\frac{1}{2},6} \right)}} = \frac{{ - \frac{16}{9}}}{ - 1} = 1.78$$

23 $$\frac{{c^{QCRE} \left( {\frac{1}{2},\frac{5}{6},8} \right)}}{{c^{QCRE} \left( {\frac{1}{3},\frac{2}{3},8} \right)}} = \frac{{\frac{25}{36}}}{{\frac{16}{81}}} = 3.52$$

### Solving inter-ring distances optimization problem for the TCRE configuration

**Fig. 4:**
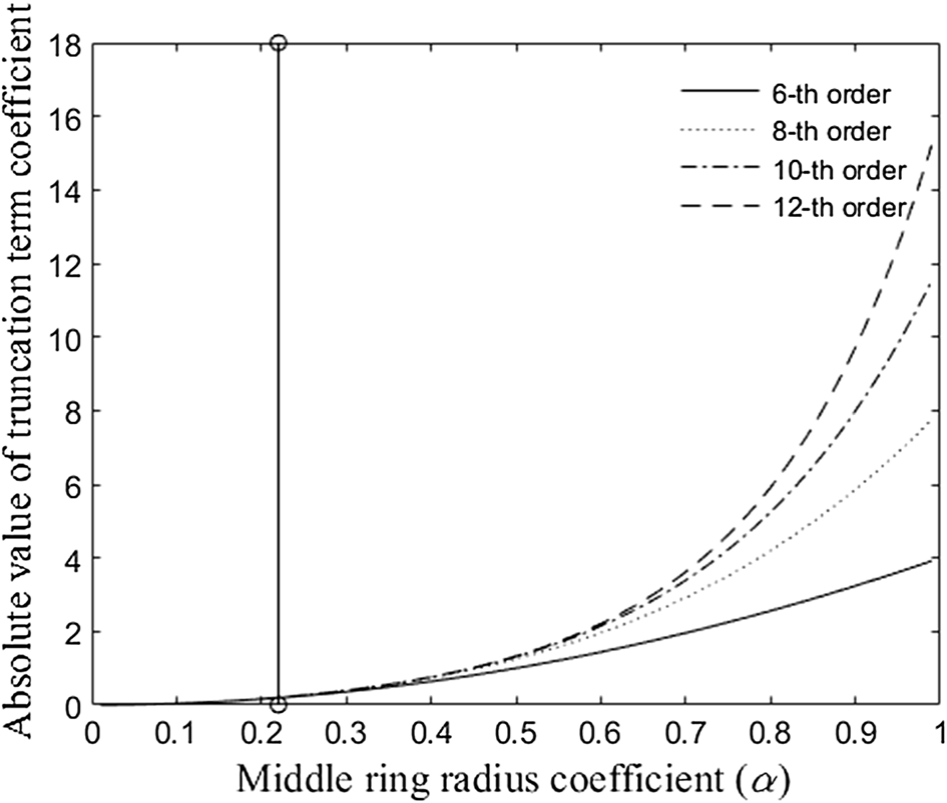
Relationship between the absolute values of truncation term coefficients and middle ring radius coefficient *α* for the TCRE configuration along with the 5th percentile boundary value *α* = 0.22

Relationship between the absolute values of truncation term coefficients and middle ring radius coefficient *α* based on the function *c*^*TCRE*^(*α*, *k*) for TCRE configuration and truncation term order *k* ranging from 6 to 12 is presented in Fig. 4. As described in “Methods” section, the 5th percentile (corresponding to the absolute value of the truncation term coefficient equal to 0.2) was used to determine the boundary value of *α* for the lowest nonzero truncation term order equal to 6 and resulting in *α* = 0.22. Therefore, the optimal range of distances between the central disc and the middle concentric ring of radius *αr* that keeps absolute values of the sixth order truncation term coefficients within the 5th percentile is determined by inequality 0 < *α* ≤ 0.22.

### Solving inter-ring distances optimization problem for the QCRE configuration

**Fig. 5:**
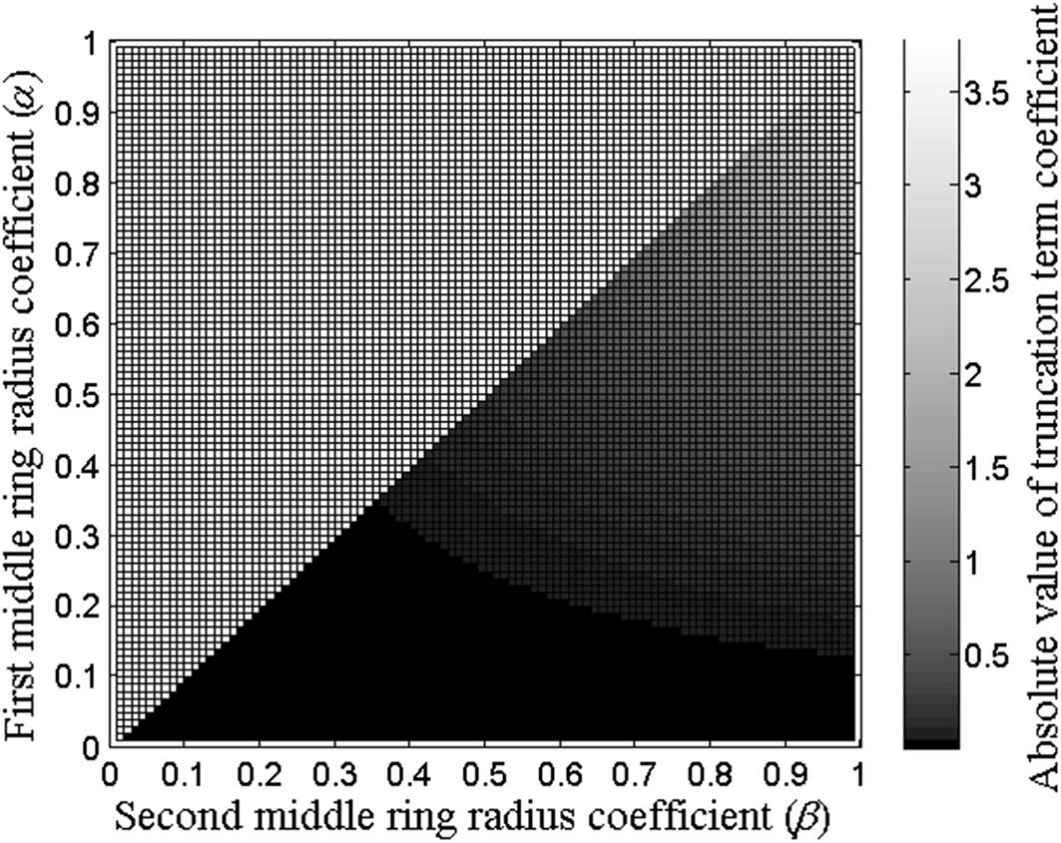
Absolute values of truncation term coefficients for the first and the second middle ring radii coefficients *α* and *β* and truncation term order *k* equal to 8 for the QCRE configuration

Absolute values of truncation term coefficients based on the function *c*^*QCRE*^(*α*, *β*, *k*) for all the combinations of the first middle ring radius coefficient *α* and the second middle ring radius coefficient *β* that satisfy 0 < *α *<* β *<1 for QCRE configuration and the lowest nonzero truncation term order *k* equal to 8 are presented in Fig. 5.

**Fig. 6:**
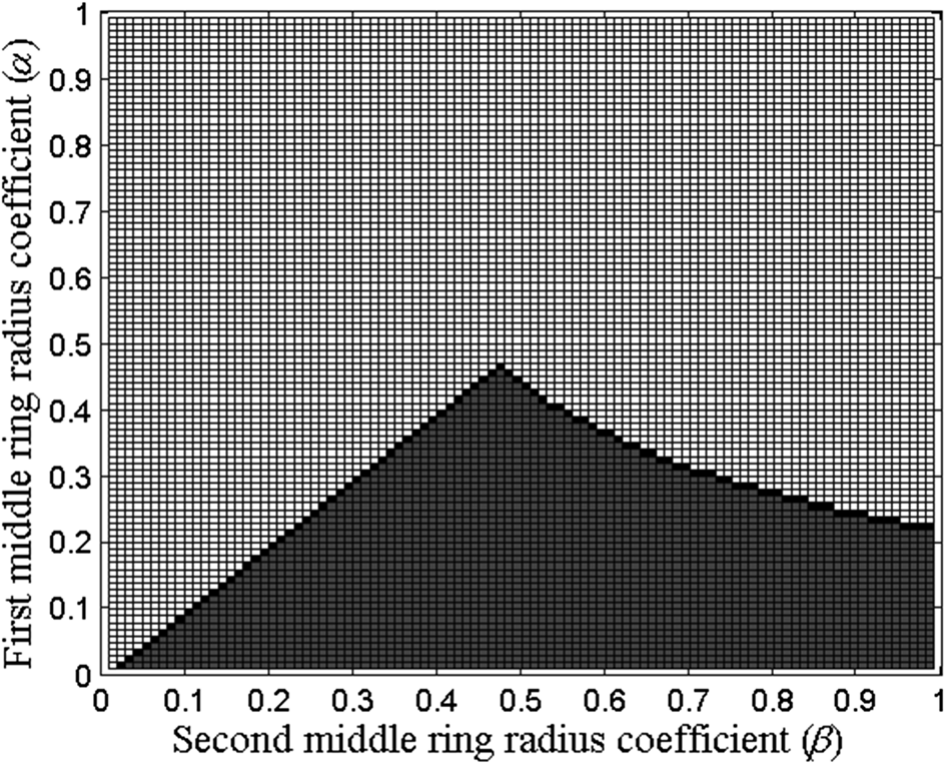
Absolute values of truncation term coefficients within the 5th percentile (gray) along with the boundary (black) separating them from the values outside of the 5th percentile for the first and the second middle ring radii coefficients *α* and *β*

As described in “Methods” section, the 5th percentile (corresponding to the absolute value of the truncation term coefficient equal to 0.19) was used to find the boundary values of *α* and *β* that determine the optimal range of distances between the central disc and both middle concentric rings with radii *αr* and *βr* respectively which keeps absolute values of the eighth order truncation term coefficients within the 5th percentile as presented in Fig. 6.

**Fig. 7:**
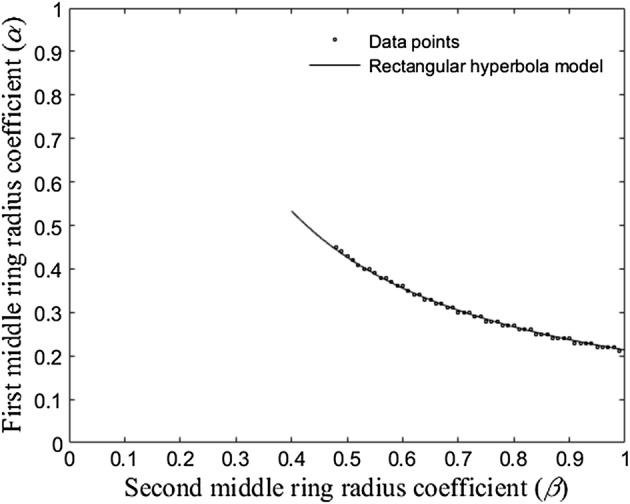
Absolute values of truncation term coefficients with rectangular hyperbola model (*m* = 0.21) fitted to the data points

While the linear portion of the boundary in Fig. 6 is described by the inequality *α* < *β*, the nonlinear portion had to be fitted with a curve first. Based on the shape of the nonlinear portion of the boundary, a rectangular hyperbola model had been chosen [26]. Even the simplest rectangular hyperbola model *α* = *m*/*β*, where *m* is a real constant, provides a good fit to our data presented in Fig. 7 for *m *= 0.21. Goodness-of-fit metric R-squared indicates that the model fit explained 99.79% of the total variation in the data [25].

Therefore, the optimal range of distances between the central disc and the first and the second middle concentric rings with radii *αr* and *βr* that keeps absolute values of the eighth order truncation term coefficients within the 5th percentile is determined by two inequalities 0 < *α *<* β *<1 and *α* ≤ 0.21/*β* or, equivalently, *αβ* ≤ 0.21.

### FEM modeling

**Fig. 8:**
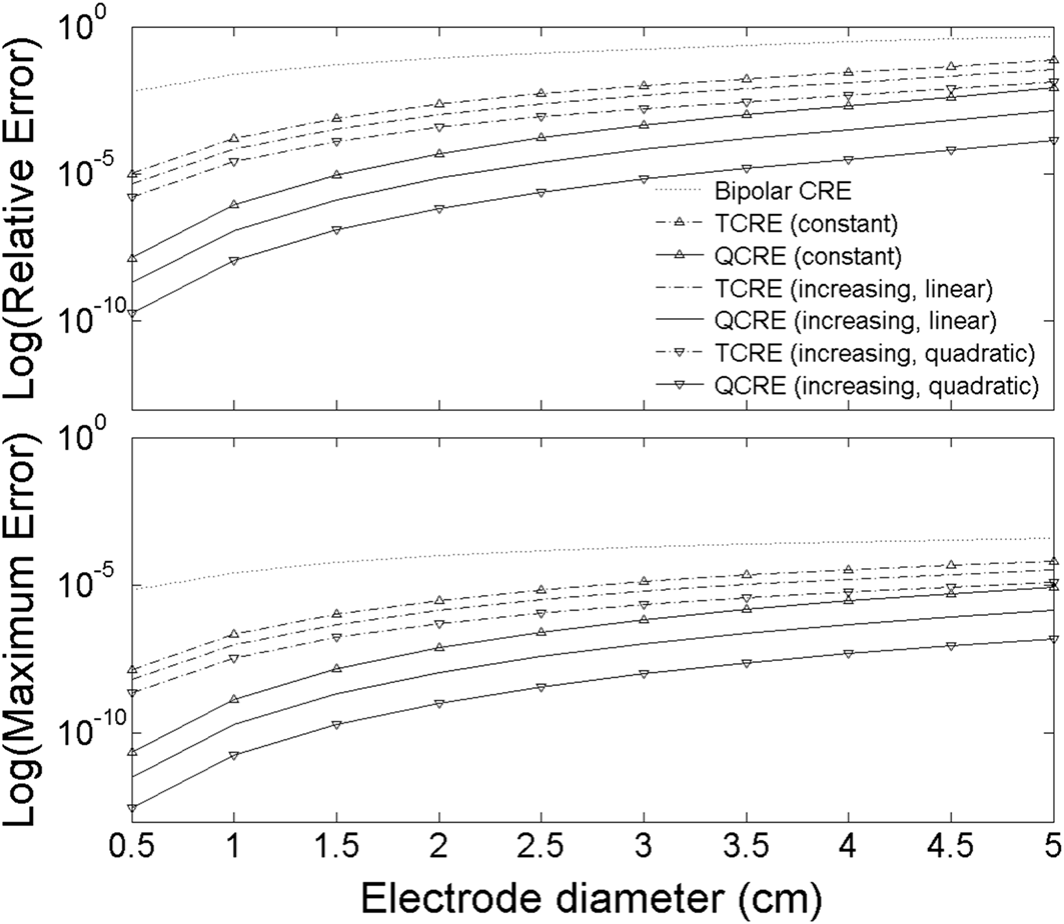
Relative (top panel) and Maximum (bottom panel) Errors for seven Laplacian estimates corresponding to bipolar CRE, TCRE, and QCRE configurations

FEM modeling results for the two error measures computed for seven CRE configurations using Eqs. (18) and (19) are presented on a semi-log scale in Fig. 8 for CRE diameters ranging from 0.5 to 5 cm.

Figure 8 suggests that novel quadratically increasing inter-ring distances TCRE and QCRE configurations hold potential for an improvement in Laplacian estimation errors over previously proposed constant [17] and linearly increasing [18] inter-ring distances counterparts. Moreover, improvement appears to become more significant with the increase of the number of rings (i.e. there is more improvement for the QCRE configuration in comparison with the TCRE one). This stems from comparison of averages (mean ± standard deviation for 10 different sizes of each CRE configuration) of errors for linearly increasing inter-ring distances and quadratically increasing inter-ring distances CREs. Compared to their quadratically increasing inter-ring distances counterparts Relative and Maximum Errors are 2.73 ± 0.04 and 2.72 ± 0.05 times higher on average for linearly increasing inter-ring distances TCREa and 10.32 ± 0.3 and 10.23 ± 0.32 times higher on average for linearly increasing inter-ring distances QCREs respectively (Fig. 8).

These ratios of Relative and Maximum Errors involving the novel quadratically increasing inter-ring distances CREs were compared to analytic ratios of truncation term coefficients using Eqs. (13) and (17) respectively. For quadratically increasing inter-ring distances TCRE and QCRE configurations we have truncation term coefficient functions $c^{TCRE} \left( {\frac{1}{5},k} \right)$ and $c^{QCRE} \left( {\frac{1}{14},\frac{5}{14},k} \right)$ respectively. The analytic ratios of truncation term coefficients for linearly increasing over quadratically increasing inter-ring distances TCRE and QCRE configurations calculated for the lowest nonzero truncation term orders equal to 6 and 8 respectively and rounded to the nearest hundredth are equal to:

24 $$\frac{{c^{TCRE} \left( {\frac{1}{3},6} \right)}}{{c^{TCRE} \left( {\frac{1}{5},6} \right)}} = \frac{{ - \frac{4}{9}}}{{ - \frac{4}{25}}} = 2.78$$

25 $$\frac{{c^{QCRE} \left( {\frac{1}{6},\frac{1}{2},8} \right)}}{{c^{QCRE} \left( {\frac{1}{14},\frac{5}{14},8} \right)}} = \frac{{\frac{1}{36}}}{{\frac{25}{9604}}} = 10.67$$

Consistent with the comparison between linearly decreasing, constant, and linearly increasing inter-ring distances CREs from [18], the FEM derived ratios of Relative and Maximum Errors involving the novel quadratically increasing inter-ring CREs are comparable (difference of less than 5%) to the respective analytic ratios of truncation term coefficients from Eqs. (24) and (25).

ANOVA results assessing the effect of factors A (inter-ring distances), B (CRE diameter), and C (number of rings) along with the effect of all possible two-factor interactions on Relative and Maximum Errors suggest that all three factors are statistically significant (Relative Error: df = 9, *F* = 85.76, *p* < 0.0001; Maximum Error: df = 9, *F* = 129.90, *p* < 0.0001) for the optimal transform being natural logarithmic function (**λ** = 0 for both the Relative Error and the Maximum Error) as determined using the Box–Cox procedure [25]. Individual effects of the three factors are: A (Relative Error: df = 2, *F* = 32.42, *p* < 0.0001; Maximum Error: df = 2, *F* = 55.87, *p* < 0.0001), B (Relative Error: df = 1, *F* = 251.24, *p* < 0.0001; Maximum Error: df = 1, *F* = 311.89, *p* < 0.0001), and C (Relative Error: df = 1, *F* = 427.55, *p* < 0.0001; Maximum Error: df = 1, *F* = 422.95, *p* < 0.0001). Out of the three two-factor interactions assessed none had statistically significant effect for both response variables.

## Discussion

This paper continues our work toward improving the accuracy of Laplacian estimation via multipolar CREs derived using the (4*n* + 1)-point method proposed in [17] and modified for linearly variable inter-ring distances CREs in [18]. Prior to [18], inter-ring distances of a CRE were not considered to be a means of improving the accuracy of Laplacian estimation with, to the best of the author’s knowledge, all the previous CRE research having been based on assumption of constant inter-ring distances.

This research direction is important since ability to estimate the Laplacian at each electrode constitutes the primary biomedical significance of CREs. Further improvement of the accuracy of Laplacian estimation via optimized inter-ring distances CREs may contribute to the advancement of noninvasive electrophysiological electrode design with application areas not limited to EEG, ECG, EMG, etc. In particular, for the case of EEG, since “negative Laplacian is approximately proportional to cortical (or dura) surface potential” [27] and enhances the high spatial frequency components of the brain activity close to the electrode [28], Laplacian filtering has been proven to be a high-pass filter for cortical imaging [29, 30]. Ability to attenuate distant sources sharply is critical for location specific EEG applications such as brain–computer interface, seizure onset detection, and detection of high-frequency oscillations and seizure onset zones which is why superiority of tEEG via TCRE over EEG via conventional disc electrodes has been recently shown in these areas [4–9]. This superiority depends on the ability to estimate the surface Laplacian as accurately as possible which is why every application currently recording and utilizing surface Laplacian signals such as tEEG may benefit from more accurate Laplacian estimation. Therefore, this paper provides an innovative solution (ability to optimize the inter-ring distances of the CRE) to improve the accuracy of an acquired signal (surface Laplacian estimate) via improved design of the sensor (such as the novel quadratically increasing inter-ring distances design) selected from the class of all the optimized inter-ring distances designs defined by the solutions of the proposed general inter-ring distances optimization problem. This work may provide insight for future sensor design in noninvasive electrophysiological measurement systems that use CREs to acquire electrical signals such as from the brain, intestines, heart or uterus for diagnostic purposes [4–16].

The contribution of this paper is threefold. First, analytic ratios of truncation term coefficients for linearly increasing, linearly decreasing, and constant inter-ring distances TCRE and QCRE configurations from [18] were recalculated using truncation term coefficient functions derived for the proposed general inter-ring distances optimization problem in order to validate those functions. In [18] it has been shown that these analytic ratios are comparable (difference of less than 5%) to the respective ratios of Relative and Maximum Errors of Laplacian estimation computed using the FEM model. Therefore, it was important to integrate this relationship between analytic and FEM results established in [18] into the framework of the proposed general inter-ring distances optimization problem for the (4*n* + 1)-point method of Laplacian estimation since it allows quantifying the expected improvement in FEM Laplacian estimation accuracy analytically. Furthermore, an identical result was obtained for ratios involving the novel quadratically increasing inter-ring distances TCRE and QCRE configurations proposed in this study.

Second, the general inter-ring distances optimization problem has been solved for TCRE and QCRE configurations. The same approach can be applied to solve corresponding problems for higher numbers of concentric rings in pentapolar, sextapolar, etc. CRE configurations even though the number of decision variables will increase by one for each additional concentric ring. This is a fundamental improvement over preliminary work such as [17] where just constant inter-ring distances have been considered and [18] where only two specific cases of linearly variable inter-ring distances were proposed and assessed in that it allows to further improve the surface Laplacian estimation accuracy via optimized inter-ring distances CREs. As was hypothesized in [18], solutions of the general inter-ring distances optimization problem correspond to nonlinear relationships between inter-ring distances as opposed to the linear relationship considered in [18].

For the TCRE configuration, the optimal range of distances between the central disc and the middle concentric ring of radius *αr* that keeps absolute values of the sixth order truncation term coefficients within the 5th percentile was determined by inequality 0 < *α* ≤ 0.22. Currently used constant inter-ring distances TCREs [1–9] correspond to *α* = 0.5 while linearly increasing and linearly decreasing inter-ring distances TCREs from [18] correspond to *α* = 0.33 and *α* = 0.67 respectively rounded to the nearest hundredth. Therefore, all three previously considered TCRE configurations fall outside the 5th percentile range corresponding to optimized inter-ring distances. For the QCREs configuration, the optimal range of distances between the central disc and the first and the second middle concentric rings with radii *αr* and *βr* respectively that keeps absolute values of the eighth order truncation term coefficients within the 5th percentile is determined by two inequalities 0 < *α *<* β *<1 and *αβ *≤ 0.21. Constant inter-ring distances QCREs correspond to *α* = 0.33 and *β* = 0.67 while linearly increasing and decreasing inter-ring distances QCREs from [18] correspond to *α* = 0.17 and *β* = 0.5 and *α* = 0.5 and *β* = 0.83 respectively rounded to the nearest hundredth. Therefore, out of three previously considered QCRE configurations only linearly increasing inter-ring distances configuration falls within the 5th percentile range corresponding to optimized inter-ring distances. For the novel quadratically increasing inter-ring distances CREs proposed in this paper both TCRE (*α* = 0.2) and QCRE (*α* = 0.07 and *β* = 0.36) configurations fall within the 5th percentile range corresponding to optimized inter-ring distances.

Finally, full factorial ANOVA was used to confirm the statistical significance of FEM results obtained for CRE configurations including the optimized quadratically increasing inter-ring distances CREs. The ANOVA results for comparison of surface Laplacian estimates corresponding to different CRE configurations showed statistical significance of all three factors included in the study. It was important to confirm that the accuracy of Laplacian estimation increases (Relative and Maximum Errors decrease) with an increase in the number of rings *n* (factor B) and decreases (Relative and Maximum Errors increase) with an increase of the CRE diameter (factor C), which is consistent with the ANOVA results obtained in [17, 20]. However, the most important ANOVA result obtained was that, for the case of inter-ring distances (factor A), the Laplacian estimates for novel quadratically increasing inter-ring distances CREs are significantly more accurate than the ones for their constant and linearly increasing inter-ring distances counterparts (*p* < 0.0001). In particular, more than two- and tenfold decreases in estimation error are expected for optimized quadratically increasing inter-ring distances TCREs and QCREs respectively compared to corresponding linearly increasing inter-ring distances CRE configurations from [18]. This result further suggests the potential of using the distances between the rings as a means of improving the accuracy of surface Laplacian estimation via CREs.

Directions of future work are twofold. The first one is based on the limitation of the (4*n* + 1)-point method. At this point of time the widths of concentric rings and the radius of the central disc are not taken into account and therefore cannot be optimized. Moreover, assuming these parameters to be negligible is inconsistent with the design of currently used TCREs (Fig. 1b). In order to pursue the ultimate goal of optimizing all of the CRE parameters simultaneously, the first direction is to include these parameters into future modifications of the (4*n* + 1)-point method along with the currently included number of rings and inter-ring distances. The first step in this direction has been taken in [31] by deriving a Laplacian estimate for a proof of concept TCRE with incorporated radius of the central disc and the widths of the concentric rings. However, it remains unclear how this proof of concept could be practically incorporated into a modification of the (4*n* + 1)-point method and/or used for design optimization purposes due to associated increases in complexity of the linear algebra involved and in the number of decision variables in the optimization problem.

The second direction is to build prototypes of optimized inter-ring distances CREs and assess them on real life data: phantom, animal model, and human. These prototypes will allow quantifying the translation of truncation error of Laplacian estimation assessed in this paper into improvement of spatial selectivity, signal-to-noise ratio, source mutual information, etc. the same way it has been quantified for tEEG via TCREs compared to EEG with conventional disc electrodes in [3]. The first step in this direction has been taken in [19] by assessing stencil printed TCRE prototypes closely resembling the linearly increasing inter-ring distances design proposed in [18] on human EEG, ECG, and EMG data with obtained results suggesting enhanced spatial resolution and localization of signal sources. To the best of the author’s knowledge these are the first physical prototypes of variable inter-ring distances CREs and they stemmed from the analytical and modeling results in [18]. Next, prototypes of optimized inter-ring distances CRE designs such as the quadratically increasing inter-ring distances TCREs and QCREs proposed in this paper are needed. These prototypes need to be compared directly to their constant and linearly increasing inter-ring distances counterparts in addition to comparison against the conventional disc electrodes drawn in [19]. Moreover, the question of how small can the distances between concentric rings become without partial shorting due to salt bridges becoming a significant factor affecting the Laplacian estimation can be answered using physical CRE prototypes as well. If prototype assessment results would suggest that physical considerations render the inter-ring distances within the 5th percentile region impractical, then inter-ring distances within the higher percentile region will be considered such as, for example, the 10th percentile region resulting in 0 < *α* ≤ 0.31 for the TCRE configuration and 0 < *α *<* β *<1 and *αβ *≤ 0.3 for the QCRE configuration.

## Conclusions

As noninvasive tripolar concentric ring electrodes are gaining increased recognition in a range of applications related to electrophysiological measurement due to their unique capabilities this paper establishes a theoretical basis for optimization of variable inter-ring distances in concentric ring electrode design. Previous findings for constant and linearly variable inter-ring distances electrode configurations are integrated into the framework of the general inter-ring distances optimization problem. The problem is solved for tripolar and quadripolar concentric ring electrode configurations and solutions, in the form of optimal ranges for inter-ring distances, may offer more accurate surface Laplacian estimates for electrophysiological measurement systems based on optimized inter-ring distances concentric ring electrodes. Full factorial analysis of variance is used to assess finite element method modeling results obtained for concentric ring electrode configurations including the optimized inter-ring distances ones. It showed statistical significance of the effect of three factors included in this study on the estimation accuracy of surface Laplacian including the inter-ring distances suggesting the potential of using optimization of inter-ring distances to improve the concentric ring electrode design.

## Abbreviations

CRE: concentric ring electrode
EEG: electroencephalography
TCRE: tripolar concentric ring electrode
FPM: five-point method
tEEG: Laplacian electroencephalography via tripolar concentric ring electrode
ECG: electrocardiography
FEM: finite element method
QCRE: quadripolar concentric ring electrode
EMG: electromyography
ANOVA: analysis of variance

## References

[1] Besio WG, Koka K, Aakula R, Dai W (2006). Tri-polar concentric ring electrode development for Laplacian electroencephalography. IEEE Trans Biomed Eng.

[2] Besio WG, Aakula R, Koka K, Dai W (2006). Development of a tri-polar concentric ring electrode for acquiring accurate laplacian body surface potentials. Ann Biomed Eng.

[3] Koka K, Besio WG (2007). Improvement of spatial selectivity and decrease of mutual information of tri-polar concentric ring electrodes. J Neurosci Methods.

[4] Besio WG, Cao H, Zhou P (2008). Application of tripolar concentric electrodes and prefeature selection algorithm for brain–computer interface. IEEE Trans Neural Syst Rehabil Eng.

[5] Boudria Y, Feltane A, Besio W (2014). Significant improvement in one-dimensional cursor control using Laplacian electroencephalography over electroencephalography. J Neural Eng.

[6] Makeyev O, Liu X, Luna-Munguia H, Rogel-Salazar G, Mucio-Ramirez S, Liu Y (2012). Toward a noninvasive automatic seizure control system in rats with transcranial focal stimulations via tripolar concentric ring electrodes. IEEE Trans Neural Syst Rehabil Eng.

[7] Feltane A, Boudreaux-Bartels GF, Besio WG (2012). Automatic seizure detection in rats using laplacian eeg and verification with human seizure signals. Ann Biomed Eng.

[8] Besio WG, Martinez-Juarez IE, Makeyev O, Gaitanis JN, Blum AS, Fisher RS (2014). High-frequency oscillations recorded on the scalp of patients with epilepsy using tripolar concentric ring electrodes. IEEE J Transl Eng Health Med..

[9] Makeyev O, Musngi M, Lee F, Tamayo M (2017). Recent Advances in high-frequency oscillations and seizure onset detection using laplacian electroencephalography via tripolar concentric ring electrodes. Proceedings..

[10] Prats-Boluda G, Garcia-Casado J, Martinez-de-Juan JL, Ye-Lin Y (2011). Active concentric ring electrode for non-invasive detection of intestinal myoelectric signals. Med Eng Phys.

[11] Garcia-Casado J, Zena-Gimenez V, Prats-Boluda G, Ye-Lin Y (2013). Enhancement of non-invasive recording of electroenterogram by means of a flexible array of concentric ring electrodes. Ann Biomed Eng.

[12] Besio WG, Chen T (2007). Tripolar Laplacian electrocardiogram and moment of activation isochronal mapping. Physiol Meas.

[13] Prats-Boluda G, Ye-Lin Y, Garcia-Breijo E, Ibañez J, Garcia-Casado J (2012). Active flexible concentric ring electrode for non-invasive surface bioelectrical recordings. Meas Sci Technol.

[14] Prats-Boluda G, Ye-Lin Y, Bueno-Barrachina JM, de Sanabria RR, Garcia-Casado J (2016). Towards the clinical use of concentric electrodes in ECG recordings: influence of ring dimensions and electrode position. Meas Sci Technol.

[15] Ye-Lin Y, Bueno-Barrachina JM, Prats-boluda G, Rodriguez de Sanabria R, Garcia-Casado J (2017). Wireless sensor node for non-invasive high precision electrocardiographic signal acquisition based on a multi-ring electrode. Measurement.

[16] Ye-Lin Y, Alberola-Rubio J, Prats-boluda G, Perales A, Desantes D, Garcia-Casado J (2014). Feasibility and analysis of bipolar concentric recording of electrohysterogram with flexible active electrode. Ann Biomed Eng.

[17] Makeyev O, Ding Q, Besio WG (2016). Improving the accuracy of Laplacian estimation with novel multipolar concentric ring electrodes. Measurement.

[18] Makeyev O, Besio WG (2016). Improving the accuracy of Laplacian estimation with novel variable inter-ring distances concentric ring electrodes. Sensors..

[19] Wang K, Parekh U, Pailla T, Garudadri H, Gilja V, Ng TN (2017). Stretchable dry electrodes with concentric ring geometry for enhancing spatial resolution in electrophysiology. Adv Healthc Mater..

[20] Makeyev O, Joe C, Lee C, Besio WG. Analysis of variance to assess statistical significance of Laplacian estimation accuracy improvement due to novel variable inter-ring distances concentric ring electrodes. In: 39th Annu Int Conf IEEE Eng Med Biol Soc. 2017. p. 4110–3. 10.1109/EMBC.2017.8037760 29060801

[21] Huiskamp G (1991). Difference formulas for the surface Laplacian on a triangulated surface. J Comput Phys.

[22] Weisstein EW. Triangular number. http://mathworld.wolfram.com/TriangularNumber.html. Accessed 11 Feb 2016.

[23] King MR, Mody NA (2010). Numerical and statistical methods for bioengineering: applications in MATLAB.

[24] Besio WG, Fasiuddin M (2005). Quantizing the depth of bioelectrical sources for non-invasive 3D imaging. J Bioelectromagn..

[25] Montgomery DC (2008). Design and analysis of experiments.

[26] Wohlfart B, Edman KAP (1994). Rectangular hyperbola fitted to muscle force-velocity data using three-dimensional regression analysis. Exp Physiol.

[27] Tong S, Thakor NV (2009). Quantitative EEG analysis methods and clinical applications.

[28] Babiloni F, Babiloni C, Fattorini L, Carducci F, Onorati P, Urbano A (1995). Performances of surface Laplacian estimators: a study of simulated and real scalp potential distributions. Brain Topogr.

[29] Srinivasan R (1999). Methods to improve the spatial resolution of EEG. Int J Bioelectromagn..

[30] Kramer MA, Szeri AJ (2004). Quantitative approximation of the cortical surface potential from EEG and ECoG measurements. IEEE Trans Biomed Eng.

[31] Makeyev O, Lee C, Besio WG. Proof of concept Laplacian estimate derived for noninvasive tripolar concentric ring electrode with incorporated radius of the central disc and the widths of the concentric rings. In: 39th Annu Int Conf IEEE Eng Med Biol Soc. 2017. p. 841–4. 10.1109/EMBC.2017.8036955 29060003

